# A zinc-doped endodontic cement facilitates functional mineralization and stress dissipation at the dentin surface

**DOI:** 10.4317/medoral.22751

**Published:** 2018-11-21

**Authors:** Manuel Toledano, Raquel Osorio, Mayra C. Pérez-Álvarez, Estrella Osorio, Christopher D. Lynch, Manuel Toledano-Osorio

**Affiliations:** 1 DDS, PhD, Professor. University of Granada, Faculty of Dentistry, Dental Materials Section. Colegio Máximo de Cartuja s/n, 18071 – Granada - Spain; 2 DDS, PhD, Professor. University of La Havana, Biomaterials Department. San Lázaro y L. Municipio Plaza de la Revolución. La Havana- Cuba; 3 BDS, PhD, Professor. University Dental School & Hospital/ University College Cork, Wilton, Cork – Ireland; 4 BS, Research Fellow. University of Granada, Faculty of Dentistry, Dental Materials Section. Colegio Máximo de Cartuja s/n, 18071 – Granada - Spain

## Abstract

**Background:**

The purpose of this study was to evaluate nanohardness and viscoelastic behavior of dentin surfaces treated with two canal sealer cements for dentin remineralization.

**Material and Methods:**

Dentin surfaces were subjected to: i) 37% phosphoric acid (PA) or ii) 0.5 M ethylenediaminetetraacetic acid (EDTA) conditioning prior to the application of two experimental hydroxyapatite-based cements, containing sodium hydroxide (calcypatite) or zinc oxide (oxipatite), respectively. Samples were stored in simulated body fluid during 24 h or 21 d. The intertubular and peritubular dentin were evaluated using a nanoindenter to assess nanohardness (*Hi*). The load/displacement responses were used for the nano-dynamic mechanical analysis to estimate complex modulus (E*) and tan delta (*δ*). The modulus mapping was obtained by imposing a quasistatic force setpoint to which a sinusoidal force was superimposed. AFM imaging and FESEM analysis were performed.

**Results:**

After 21 d of storage, dentin surfaces treated with EDTA+calcypatite, PA+calcypatite and EDTA+oxipatite showed viscoelastic discrepancies between peritubular and intertubular dentin, meaning a risk for cracking and breakdown of the surface. At both 24 h and 21 d, tan *δ* values at intertubular dentin treated with the four treatments performed similar. At 21 d time point, intertubular dentin treated with PA+oxipatite achieved the highest complex modulus and nanohardness, i.e., highest resistance to deformation and functional mineralization, among groups.

**Conclusions:**

Intertubular and peritubular dentin treated with PA+oxipatite showed similar values of tan *δ* after 21 d of storage. This produced a favorable dissipation of energy with minimal energy concentration, preserving the structural integrity at the dentin surface.

** Key words:**Dentin, fracture, hydroxyapatite, remineralization, viscoelastic, zinc.

## Introduction

Self-repairing dental cements, which can promote deposition of calcium phosphate at the material-tooth interface, may have a promising preventive contribution in restorative dentistry (1). New biomaterials for use in regenerative endodontics should facilitate dentin remineralization (2) without root fracture. This is not the case of calcium hydroxide [Ca(OH)2], which reduces the elastic modulus, microhardness (3) and flexural strength (4) of mineralized dentin. Fracture is one of the primary forms of restored tooth failure, as cracked teeth are prevalent in teeth receiving endodontic treatments (5). Calcium hydroxide powder in association with hydroxyapatite (HAp), has been proposed to overcome this drawback. HAp possesses low mechanical strength and fracture toughness, which is an obstacle to its application in load-bearing areas. Zn-substituted HAp has been shown to possess enhanced bioactivity and improved biological and mechanical properties of nucleated minerals for tissue engineering application. This effect make zinc attractive for use as therapeutic agent in the fields of dentin regeneration.

Foraminal and intracanal space cleanliness are necessary for root canal treatment of teeth. The irrigation procedure may be done with phosphoric acid (PA) or EDTA (ethylenediaminetetraacetic acid), but PA may damage the dentin collagen (6), while EDTA does not completely remove non-collagenous proteins from dentin. Changes in mechanical properties of the dentin caused by the action of irrigants, medication and root canal filling materials for remineralizing purposes may predispose to tooth fracture. Nano-dynamic mechanical analysis (nano-DMA) may used to evaluate viscoelastic behavior of human dentin for the underlying intertubular and peritubular dentin. Microstructure of dentin is considered as a cylindrical fiber reinforced composite, with the matrix as intertubular dentin and the reinforcements as the tubule lumens and the concomitant peritubular dentin cuff (7). Discrepancies in terms of viscoelastic properties at the different structures within the dentin, mean a risk for cracking, which opposed crack opening (7) and breakdown of this interface, as low modulus regions lead to energy concentration in relatively high elastic modulus regions (8). This may account for failures at the restored teeth, leading to the cracked tooth syndrome, that occurred when the crack promotes a stress intensity that reaches the local fracture toughness of the tissue (5). Cracks deflection, microcracking, and crack bridging have been reported in the fracture of dentin (9).

The combination of mechanical data with atomic force microscopy (AFM) appears to be a valuable tool to be applied in dentin remineralization studies (10). The aim of this study was to study the nano-mechanical changes occurring after treating the dentin surfaces with two different cleaner-conditioners (PA vs EDTA), and the application of two canal sealers (calcypatite vs oxipatite) at two time points (24 h vs 21 d). The null hypothesis that was established is that changes in mechanical properties were not produced on dentin surfaces after sealer application.

## Material and Methods

-Specimen preparation and cement application 

Forty eight human third molars without caries lesions were obtained with informed consent from donors (20-40 year of age), under a protocol approved by the Institution Review Board (139/CEIH/2016). Molars were stored at 4ºC in 0.5% chloramine T for up to 1 month before use. The storage medium was replaced weekly. A flat mid-coronal dentin surface was exposed using a hard tissue microtome (Accutom-50; Struers, Copenhagem, Denmark) equipped with a slow-speed, water-cooled diamond wafering saw (330-CA RS-70300, Struers, Copenhagen, Denmark). A 180-grit silicon carbide (SiC) abrasive paper was used to produce a clinically relevant smear layer (11). A 37% phosphoric acid gel was applied on the exposed dentin surfaces for 15 s, and copiously water rinsed for 30 s, in half of the specimens (n=24). On the other half (n=24), EDTA-treated dentin, 0.5 M was applied for 60 s, and copiously water rinsed for 30 s.

Two experimental hydroxyapatite-based cements were used: 1) calcypatite, composed by modified hydroxyapatite particles and a calcium hydroxide paste and 2) oxipatite which is a combination of the hydroxyapatite particles and zinc oxide (ZnO). Detailed description of employed chemicals and cements is provided in Table 1.

The specimens were divided into the following groups (n=6) based on the tested hydroxyapatite-based cements (calcypatite vs oxipatite) and dentin-etching procedure [phosphoric acid (PA) vs EDTA]: (i) EDTA-treated dentin (EDTA control); (ii) PA-etched dentin (PA control); (iii) EDTA+calcypatite; (iv) PA+calcypatite; (v) EDTA+oxipatite; (vi) PA+oxipatite. Half of the teeth were stored for 24 h in simulated body fluid solution (SBFS) ( Table 1), and the other half during 21 d.

**Table 1:**
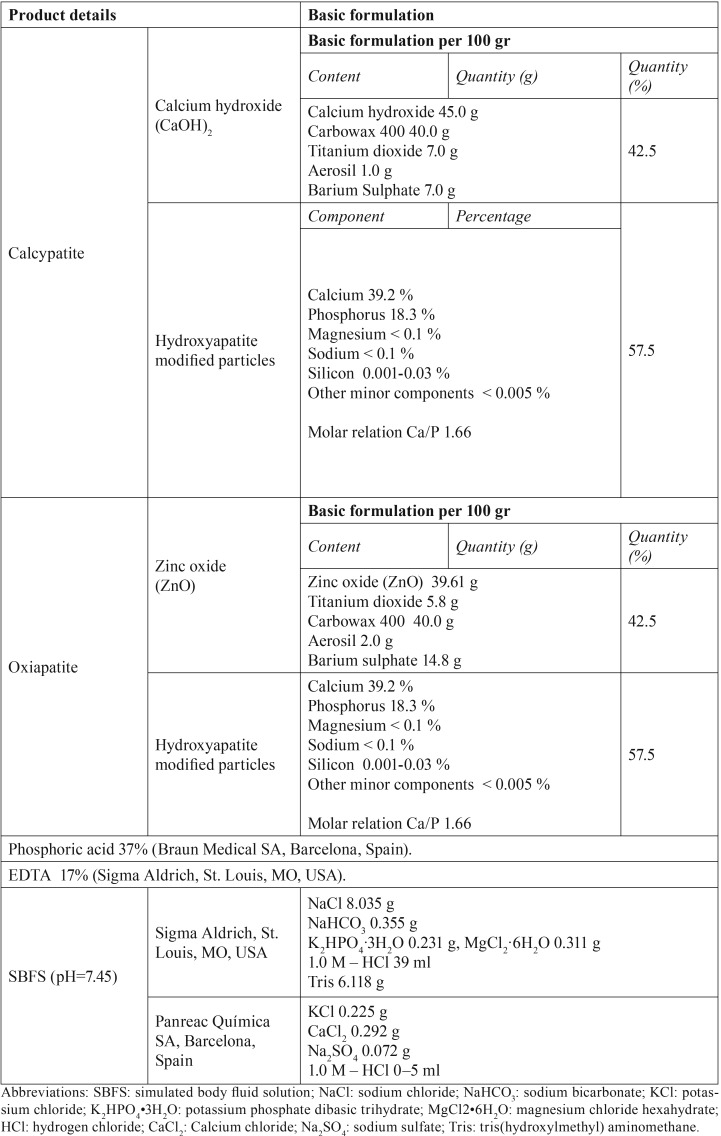
Materials and chemicals used in this study.

-Nanoindentation 

The surfaces were polished through SiC abrasive papers from 800 up to 4000 grit followed by final polishing steps performed using diamond pastes through 1 µm down to 0.25 µm (Struers LaboPol-4; Struers GmbH, Hannover, Germany). The specimens were treated in ultrasonic bath (Model QS3, Ultrawave Ltd, Cardiff, UK) containing deionized water for 5 min at each polishing step. Nanomechanical properties were assessed by means of a Hysitron Ti Premier nanoindenter (Hysitron, Inc., Minneapolis, MN), a commercial nano-DMA package. The nanoindenter tip was calibrated against a fused quartz sample using a quasistatic force setpoint of 2 µN to maintain contact between the tip and the sample surface. For each subgroup, three slabs were tested. On each slab, five indentation lines were executed in five different mesio-distal positions along the dentin surface in a straight line. Indentations were performed with a load of 4000 nN and a time function of 10 s. Specimens were scanned in a hydrated condition. To avoid dehydration a layer of ethylene glycol over the specimen surface was applied, preventing water evaporation during a typical 25-to-30-min scanning period. The distance between each indentation was kept constant by adjusting the distance intervals in 5 (±1) µm steps. Hardness (*Hi*) data were registered in GPa.

The load, *F*, was obtained as a function of the penetration depth, *h*, of the indenter in the sample. From the slope of these load-vs.-depth curves the nanoindentation modulus was obtained by application of different theoretical models (12,13). One of these is the Oliver-Pharr method, which is based on a continuum, isotropic, homogeneous elastic contact model to determine the reduced modulus, *Er*. To measure the nanohardness of the sample, *Hi*, was defined as: Hi=F*max*/A, where F*max* is the peak load, and A is the projected contact area of the hardness impression of the indenter. Values of nanohardness were automatically calculated by using the software Triboscan Quasi version 8.4.2.0 (Hysitron, Inc). Data were analyzed by two-way ANOVA (independent factors were type of NPs and storage time) and Student-Newman-Keuls multiple comparisons (*P* < 0.05).

-Nano-DMA analysis

Same slabs of each restored tooth were submitted to nano-DMA analysis. Property mappings were conducted using a HysitronTi 950 nanoindenter (Hysitron, Inc., Minneapolis, MN) equipped with nano-DMA III, a commercial nano-DMA package. The nanoindenter tip was calibrated against a fused quartz sample using a quasistatic force setpoint of 5 µN to maintain contact between the tip and the sample surface. A dynamic (oscillatory) force of 5 µN was superimposed on the quasistatic signal at a frequency of 200 Hz. Based on a calibration-reduced modulus value of 1.1400E+03 N/mm2 for the fused quartz, the best-fit spherical radius approximation for tip was found to be 150 nm, for the selected nano-DMA scanning parameters. Modulus mapping of the samples was conducted by imposing a quasistatic force setpoint, Fq=5 µN, to which a sinusoidal force of amplitude FA=1.8 µN and frequency f=200 Hz was superimposed. Data from regions approximately 30x30 µm in size were collected using a scanning frequency of 0.2 Hz. Viscoelastic data were acquired on the different specimens and obtained from selected surface areas of the substrate using a rastering scan pattern. For each property map, 10 sets of 225 datapoints were used to obtain the median property value of a particular region of interest. That is, the 225 datapoints represent 1.47 x 1.47 = 2.15 µm2 of each 30x30 = 900 µm2 of the scan. The datapoints from 10 such non-overlapping squares were obtained for each zone at the dentin surface; thus, for each nano-DMA parameter, 30 values (3 specimens x 10 squares) were generated for each zone.

Under steady conditions (application of a quasistatic force) the indentation modulus of the tested sample, E, was obtained by application of different models that relate the indentation force, *F*, and depth, D (12,13). Complex modulus (E*) and tan delta (*δ*) values were calculated as in Toledano *et al.*, 2017 (10). During the indentation mode, single indents were introduced on either the intertubular or peritubular dentin. In this mode of evaluation the tip geometry and corresponding contact area were determined using the conventional approach with a fused silica standard sample (12). Statistical analysis was performed with ANOVA and Student Newman Keuls multiple comparisons tests. *P*<0.05 was set for significance.

-Atomic Force Microscopy analysis (AFM) imaging

An atomic force microscope (AFM Nanoscope V, Digital Instruments, Veeco Metrology group, Santa Barbara, CA, USA) was employed in this study for topography mappings. The imaging process was undertaken inside a wet cell in a fully hydrated state, using the tapping mode, with a calibrated vertical-engaged piezo-scanner (Digital Instrument, Santa Barbara, CA, USA). A 10-nm-radius silicon nitride tip (Veeco) was attached to the end of an oscillating cantilever that came into intermittent contact with the surface at the lowest point of the oscillation. Changes in vertical position of the AFM tip at resonance frequencies near 330 kHz provided the height of the images registered as bright and dark regions. 15x15 µm digital images were recorded from each dentin interface, with a slow scan rate (0.1 Hz). To facilitate dentin surfaces observation, AFM images were tilted using a specific software (NanoScope Analysis v. 1.40, Bruker Corporation, Billerica, MA, USA).

-Field Emission Scanning Electron Microscopy (FESEM) 

Representative specimens of each group were fixed in a solution of 2.5% glutaraldehyde in 0.1 mol/L sodium cacodylate buffer for 24 h, rinsed three times in 0.1 mol/L sodium cacodylate buffer. Samples were placed in an apparatus for critical point drying (Leica EM CPD 300, Wien, Austria). They were, then, sputter-coated with carbon by means of a sputter-coating Nanotech Polaron-SEMPREP2 (Polaron Equipment Ltd., Watford, UK) and observed with a field emission scanning electron microscope (FESEM Gemini, Carl Zeiss, Oberkochen, Germany) at an accelerating voltage of 3 kV.

## Results

Nanohardness (*Hi*) of dentin surfaces was influenced by the type of canal sealer (*P*<0.05), cleaner-conditioner (*P*<0.05) and by the storage time (*P*<0.05). Interactions between factors were also significant (*P*<0.05). Mean and SD of Hi at intertubular (ID) and peritubular dentin (PD) are represented in  Table 2. The highest *Hi* was achieved after 21d of storage in specimens treated with PA+oxipatite. The lowest *Hi* values was reached at 21 d time point in dentin specimens treated with EDTA+oxipatite. *Hi*, after 21 d, was higher than at 24 h in all groups, except when EDTA+oxipatite was used, at both ID and PD.

**Table 2:**
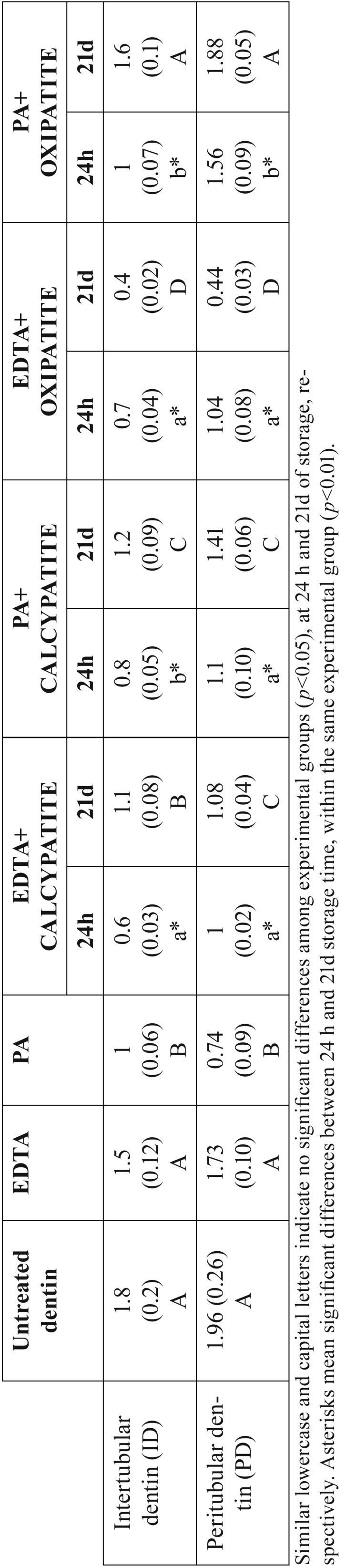
Mean and standard deviation of nanohardness values (Hi) (GPa) at dentin surfaces of the different experimental groups.

**Figure 1:**
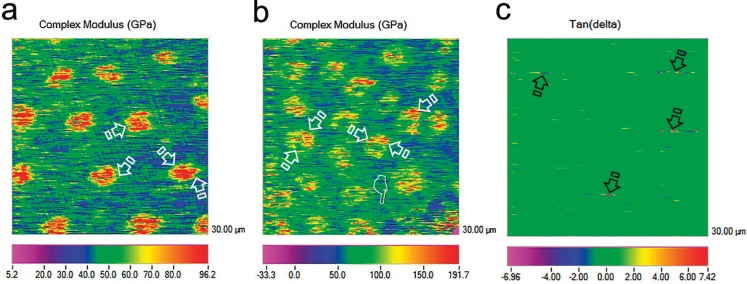
(a), Scanning mode nano-DMA analysis of the map of the complex modulus (E*) at the EDTA+oxipatite group, obtained at 21 d storing in SBFS. In the color scheme shown, the redder color corresponds to higher values of the locally measured moduli, potentially associated to E* of intratubular mineral precipitation. The intertubular dentin was represented by the blue color. Peritubular dentin was associated with the green to yellow color (arrows). (b), Scanning mode nano-DMA analysis of the map of the complex modulus (E*) at the EDTA+calcypatite group, obtained at 21 d storing in SBFS. In the color scheme shown, the redder color corresponds to higher values of the locally measured moduli, likely corresponding to the highest resistance to deformation of the peritubular dentin at both time points (arrows). E* referred to intertubular dentin appears bluish green (pointers). The pixel data array at the mapping are organized according to a E* distribution that concur with a clear delimitation between intertubular and peritubular dentin (faced arrows). (c), Scanning mode nano-DMA analysis of the map of the tan *δ* at the PA+oxipatite group, obtained at 21 d storing in SBFS. In the color scheme shown, the redder color corresponds to higher values of the locally tan *δ* value moduli, potentially associated to tan *δ* of intratubular mineral precipitation. The capacity for getting rid of the energy at peritubular dentin is represented by the blue-red-yellow diffused marks (arrows) at the mapping.

**Figure 2:**
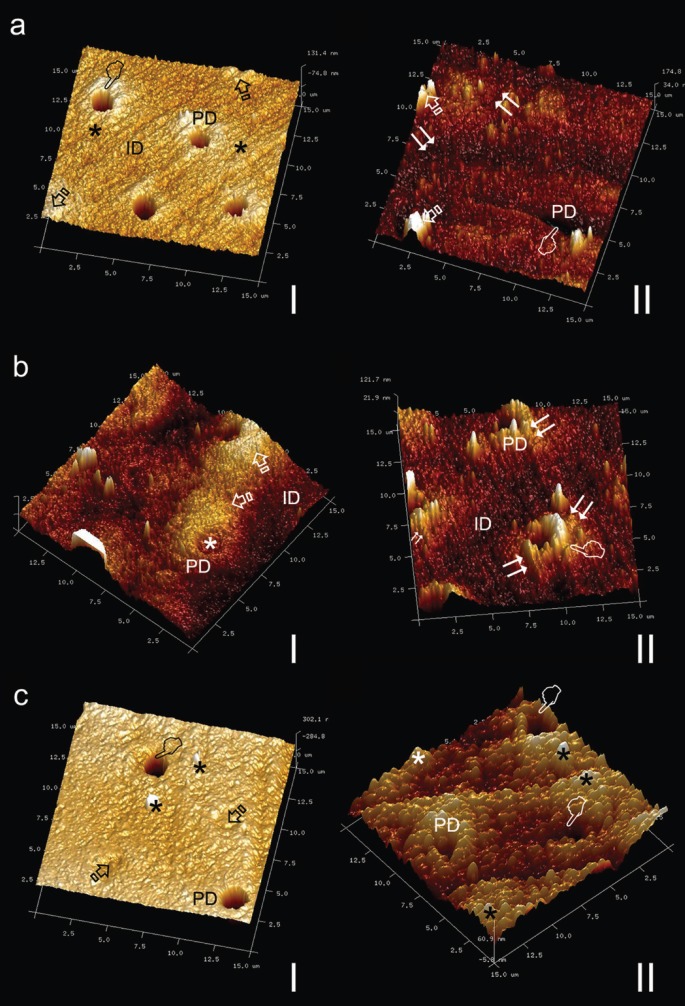
a), Topography mapping of dentin surface treated with EDTA+calcypatite obtained by AFM after 24 h (I) and 21 d (II) storing in SBFS. Peritubular (PD) and intertubular (ID) dentin are clearly observed. Some tubules appear totally occluded (arrows) or empty (pointers) at both storage times. Morphologically, homogeneous transition between peritubular and intertubular dentin (asterisks) characterizes the dentin surface at 24 h, but a neat stick-slip image crossed over the dentin surface stored 21 d time point (double arrows). b), Topography mapping of dentin surface treated with PA+calcypatite obtained by AFM after 24 h (I) and 21 d (II) storing in SBFS. Peritubular (PD) and intertubular (ID) dentin mineralization is evident. Some tubules appear partially occluded (asterisk). Morphologically, homogeneous transition between peritubular and intertubular dentin (arrows) characterizes the dentin surface at 24 h time point. Stick-slip images and little rod-like minerals (double arrows), as bridge-like structures as sight of energy dissipation were observed anchoring both peritubular and intertubular dentin, though some peritubular cuffs appeared debonded (pointer) c), Topography mapping of dentin surface treated with PA+oxipatite obtained by AFM after 24 h (I) and 21 d (II) storing in SBFS. Remarkable peritubular dentin (PD) collars are clearly defined in the intertubular dentin (ID). Tubules are totally occluded (arrows) or mineral-free (pointers). Knob-like mineral precipitates (asterisks) deposited on the dentin surface. No sign of stress concentration was discovered through the dentin surface due to the homogeneity of viscoelastic properties between peritubular and intertubular dentin, especially at the tan *δ* values.

The scanning mode nano-DMA analysis of EDTA+oxipatite, EDTA+calcypatite and PA+oxipatite are contained in Fig. 1. Fig. 2 shows topographic mapping obtained with AFM from dentin surfaces treated with EDTA+calcypatite, PA+calcypatite and PA+oxipatite, at 24 h (I) and 21 d (II) of storage time.

At intertubular dentin, samples treated with PA+oxipatite attained the highest complex modulus (E*) among groups, at 21 d storage. Tan delta (*δ*) values performed similar among groups, at 21 d ( Table 2). E* increased over time in all groups, except when EDTA+oxipatite (Fig. 1a) was used, which decreased (*p*<0.05). Storage time did not influence tan delta (*δ*), except when PA+calcypatite was used, which decreased (*p*<0.05). Viscoelastic properties of the control groups did not change over time.

At peritubular dentin, PA-treated samples showed grea-ter E* at 21 d than at 24 h of storage. Phosphoric acid etching promoted an increase of E* over time (*p*<0.05). Samples treated with EDTA+oxipatite showed lower E* at 21 d, and E* did not change when EDTA+calcypatite was applied ( Table 3) (Fig. 1b). At 21 d of storage, tan delta (*δ*) achieved the biggest values after treating dentin samples with PA+oxipatite (Fig. 1c). When dentin was treated with EDTA+oxipatite, tan delta (*δ*) decreased after 21 d of storage (*p*<0.05). Viscoelastic properties of the control groups did not change over time.

**Table 3:**
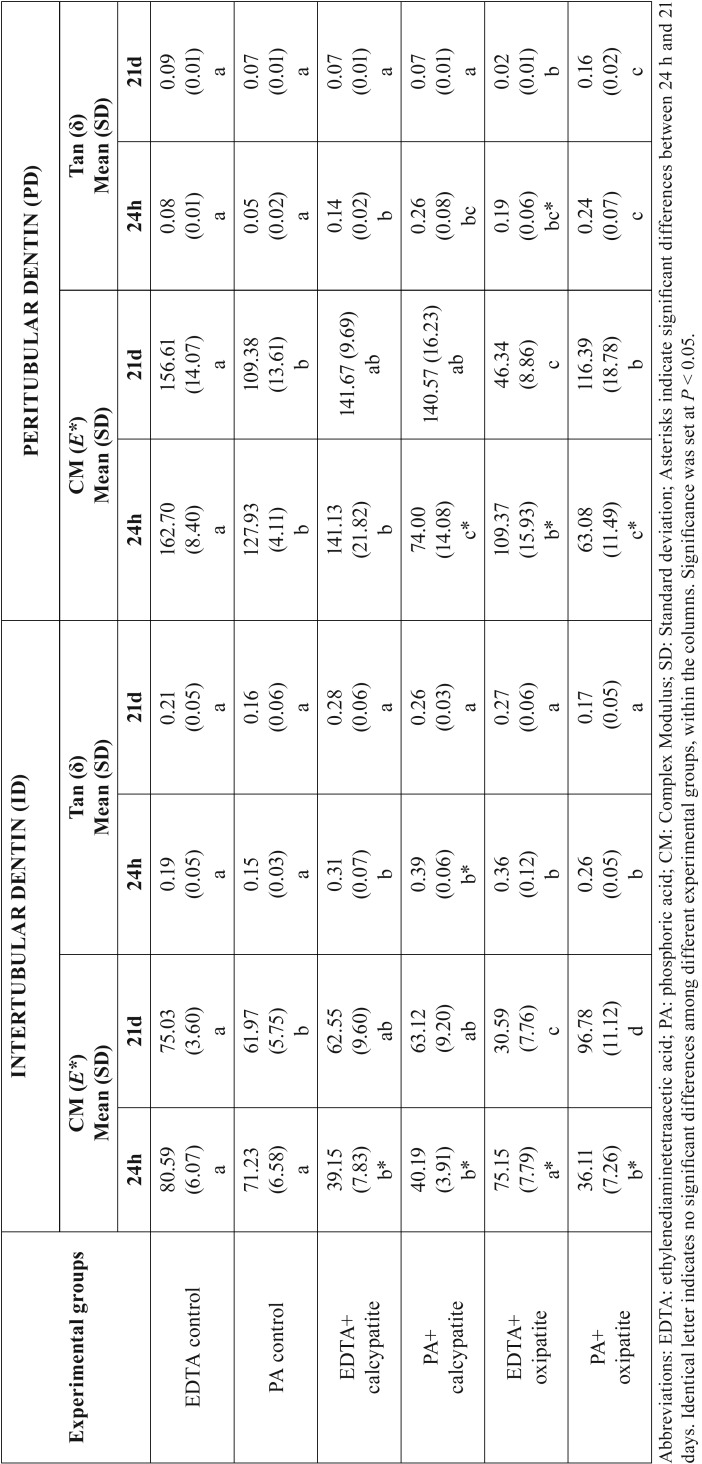
Complex modulus (GPa) and Tan (δ), at experimental dentin surfaces.

Dentin samples treated with any cement produced mineral deposits at both intertubular and peritubular dentin. Some dentinal tubules appeared occluded, at times. Strong processes of precipitated minerals covered, occasionally, the dentin surface. They concentrated, mostly, at the peritubular-intertubular dentin border (Figs. 2a•II, 2b•II, 3a). These mineral processes were absent in dentin treated with PA+oxipatite (Figs. 2c•II, 3b).

**Figure 3:**
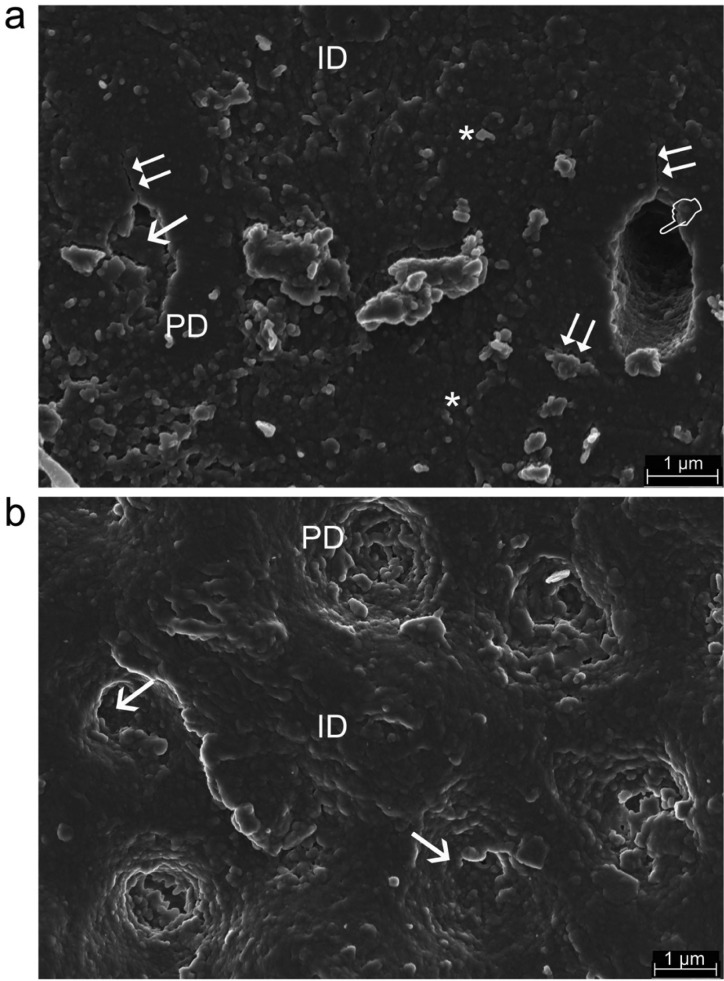
a) Representative FESEM topographic images of treated dentin with EDTA+oxipatite after 21 d storing in SBFS. Mineral deposition is completely covering the dentin surface (asterisks). Partially filled (arrow) or totally empty (pointer) tubules were shown. Crack extending through the peritubular dentin (PD) and at the peritubular/intertubular (ID) dentin limits can be observed (double arrows). b) Representative FESEM topographic images of treated dentin with phosphoric acid (PA)+oxipatite after 21 d storing in SBFS. Dentin resulted totally mineralized at both peritubular (PD) and intertubular (ID) dentin and the mineral formations did allow the display of the entrance of tubules, most of them occluded (arrows). Any peritubular cuff appeared debonded.

## Discussion

Two experimental HAp-based cements were compared in this study, (i) calcypatite (composed by modified HAp particles and a calcium hydroxide-based paste), and (ii) oxipatite (a combination of the HAp particles, and zinc oxide). The general increase of Hi at dentin surface when PA+oxipatite was applied and assessed after 21 d of storage ( Table 2) may be correlated, in the present research, with a remineralizing effect (10) linked to mineral precipitation (Ca, P and Zn) within the demineralized organic matrix. It has been previously stated that nanohardness recovery at the dentin substrate is only produced if functional and intrafibrillar remineralization occurred (14,15).

Dentin surfaces treated with calcypatite, regardless the type of acid etching (PA or EDTA), increased the resistance to deformation (E*), and thus mineralization at intertubular and peritubular dentin ( Table 3) (Fig. 2a•II), after 21 d of storage. This higher complex modulus correlates well with greater stiffness (16). When samples were treated with EDTA+calcypatite and analyzed at 21 d time point (Fig. 1b), the 2-D contour mapping of the complex modulus reflected a homogenized distribution of yellow-red color rings that corresponded with the viscoelastic behavior of peritubular dentin, with high E* (~140 GPa). They appeared surrounded by a blue greenish plateau of lower E* (~60 GPa) associated to intertubular dentin. Low modulus regions lead to stress concentration in relatively high elastic modulus regions (8). Scanning of interfaces enabled identification of both peritubular and intertubular dentin in the property maps as one of the most crucial junctions in preventing crack generation and propagation across the boundary between the two different phases (17). This mechanism would be consistent with dentin structure affectation, preferentially at this limit. These microcraks contribute to the damage of the quality parameters of the dentin (18), though dentin exhibits a rising crack growth resistance with crack extension due to its hierarchical microstructure (5).

The topography mapping obtained by AFM confirmed the existence of a neat stick-slip image crossing over the dentin surface treated with EDTA+calcypatite (Fig. 2a•II) and PA+calcypatite (Fig. 2b•II) at 21 d time point. It is speculated that these precipitated minerals, located at the peritubular-intertubular dentin limit, may be assumed as frictional pullout with cracks originated as a consequence of discrepant viscoelastic properties between both structures. Fatigue cracks in restored teeth are typically oriented perpendicular to the dentin tubules (19). Moreover, crack bridging by the collagen fibrils could enhance the toughness along direction parallel to the tubule axes (20). Cracks extending parallel to the tubules (Fig. 2b•II) experienced the highest degree of bridging and gives rise to the larger fracture toughness for this orientation (19). Calcypatite contains calcium hydroxide [Ca(OH)2] ( Table 1). Below the first zone of necrosis that results after its application on dentin, Holland, 1971 (21) described the existence of mineralized tissue resembling a bridge, composed of calcium salts and calcium-protein complexes from around 7th to the 10th day following application. The bridge also appeared in the walls of dentinal tubules or even beyond these structures (21). These mineral bridges or asperities enhance the interface strength in biological materials (22). Comparable morphological results were obtained in the present study, where it is speculated that an extended layer of new mineral with frictional pullout crossed over a part of the dentin surface (Fig. 2b•II). Nevertheless, this calcified barrier is porous and weakens roots when placed for extended periods. These observations could also suggest that there is relatively weak bonding between the peritubular and intertubular components (23) in samples treated with calcypatite, as evident from debonded peritubular cuffs in Fig. 2b•II.

Dentin surfaces treated with EDTA+oxipatite showed a ~13.5 fold lower tan *δ* values at peritubular dentin than at intertubular dentin, after 21 d of storage time ( Table 3). Tan *δ* reflects how well a material can get rid of the energy. The lower tan *δ*, the greater the proportion of energy available in the system for recoil and/or failure (24). At the 2-D contour map of the tan *δ* distribution in a specimen of dentin surface treated with EDTA+oxipatite, at 21 d of storage time (Fig. 1a), it was observed that tan *δ* values of peritubular dentin decreased an approximately 93% in comparison with the intertubular dentin. This gave rise to a zone of energy concentration at this junction. Topography mapping of dentin surface treated with EDTA+oxipatite obtained by AFM permitted to observe crack formations (Fig. 3a) and multiple zones of little rod-like minerals, as bridge-like structures anchoring both type of dentin. These bridges might be influential in effectively resisting crack propagation (18), after compensating the bridging stress intensity (5).

Nevertheless, when the dentin surfaces were treated with PA+oxipatite, tan *δ* values, obtained at 21 d of storage time proved to be practically similar, 0.17 and 0.16 at intertubular and peritubular dentin, respectively ( Table 3) (Fig. 1c). Thereby, zones and signs of energy concentration did not appear at the dentin interface (Fig. 2c•II), due to the homogeneity of viscoelastic properties, and the pullout was not evident in this PA+oxipatite treated dentin, suggesting that this interface is tightly bonded (Fig. 3b). Perhaps, the higher mineralization of dentin with PA+oxipatite results in an increase of cohesion between the two constituents, thereby decreasing the relative interfacial sliding with crack growth (23). This remains to be addressed through additional study. The presence of Zn2+ in oxipatite applied on PA-treated dentin performs as Ca/P growth inhibitors (25), favoring intrafibrillar mineralization of collagen (26) linked to the raise of Hi ( Table 2). After PA demineralization, and further oxipatite application, some Ca positions in apatite could be filled by the divalent cation Zn++ (6). As binding constant of Zn is 8.7 and it is 6.8 for Ca, therefore, Zn can compete with Ca for binding even at a very low concentration. An isomorphous substitution can be obtained when Ca2+ is replaced by Zn2+ into dentin HAp (27). Son *et al.*, 2011 (28) hold that based on crystal structure theory, one can expect that if, in HAp, the radii of doped ions (Zn: 0.074 nm) are smaller than Ca (0.099 nm), it is easy to fill them in the vacancy or intersticial sites of crystal lattice. It is speculated that PA+oxipatite provoked a more favorable tan δ values than EDTA+oxipatite because EDTA promotes a lower stoichiometric hydroxyapatite, slowing down the active dentin remodeling, with decreased maturity (29) and worse mechanical properties.

Although the results further establish the importance of including zinc oxide in the chemical formulation of hydroxyapatite based cements for endodontic purposes, there are recognized limitations, e.g, differences in microstructure between dentin of the crown and root. Endodontic treatment can introduce flaws which can lead to premature failure of the tooth as a result of a catastrophic failure, or more plausible, subcritical crack growth induced by cycling-fatigue loading (30). Nevertheless, these are to the best of our knowledge the only available results from nano-DMA experiments with morphological characterization from dentin treated with Zn oxide-modified and calcium hydroxide apatite-based cements. Based on this reasoning, the null hypothesis that was established, that changes in mechanical properties were not produced on dentin surfaces after sealer application, must be rejected.

## Conclusions

1. The application of phosphoric acid prior to the oxipatite placement facilitated functional mineralization and so the absence of zones of energy concentration at the dentin surface. Both intertubular and peritubular dentin showed similar capability to rid of the accumulated energy, thus preserving the structural integrity at the dentin surface.

2. The transfer of energy has implication on the viscoelastic response of the dentin surface treated with phosphoric acid previous to calcypatite application, and after using EDTA with either canal sealer that was used. Cracks, consistent with dentin structure affectation, were generated and propagated across the boundary between the intertubular and peritubular dentin. These microcraks concurred with some stick-slip images and dentin deposition of little rod-like minerals. These findings may be interpreted as sight of energy dissipation, and contributed to the damage of the quality parameters of the dentin.
